# miRNAs in the Diagnosis and Prognosis of Skin Cancer

**DOI:** 10.3389/fcell.2020.00071

**Published:** 2020-02-28

**Authors:** Monica Neagu, Carolina Constantin, Sanda Maria Cretoiu, Sabina Zurac

**Affiliations:** ^1^Immunology Laboratory, “Victor Babeş” National Institute of Pathology, Bucharest, Romania; ^2^Doctoral School, Faculty of Biology, University of Bucharest, Bucharest, Romania; ^3^Department of Pathology, Colentina Clinical Hospital, Bucharest, Romania; ^4^Division of Cell and Molecular Biology and Histology, “Carol Davila” University of Medicine and Pharmacy, Bucharest, Romania; ^5^Department of Pathology, Faculty of Dental Medicine, “Carol Davila” University of Medicine and Pharmacy, Bucharest, Romania

**Keywords:** miRNA, cutaneous melanoma, cutaneous squamous carcinoma, cutaneous lymphoma, Merkel cell carcinoma

## Abstract

Skin cancer is, at present, the most common type of malignancy in the Caucasian population. Its incidence has increased rapidly in the last decade for both melanoma and non-melanoma skin cancer. Differential expression profiles of microRNAs (miRNAs) have been reported for a variety of different cancers, including skin cancers. Since miRNAs’ discovery as regulators of gene expression, their importance grew in the field of oncology. miRNAs can post-transcriptionally regulate gene expression, tumor initiation, development progression, and aggressiveness. Nowadays, these short regulatory RNAs are perceived as one of the epigenetic markers for the identification of new diagnostic and/or prognostic molecular markers. Moreover, as miRNAs can drive tumorigenesis, they might eventually represent new therapy targets. Some miRNAs are pleiotropic, such as miR-214, which was found deregulated in several other tumors besides skin cancers. Some others are specific for one or more skin cancer types, like miR-21 and miR-221 for cutaneous melanoma and cutaneous squamous carcinoma or miR-155 for melanoma and cutaneous lymphoma. The goal of this review was to summarize some of the main miRNA detection technologies that are used to evaluate miRNAs in tissues and body fluids. Furthermore, their quantification limits, conformity, and robustness are discussed. Aberrant miRNA expression is analyzed for cutaneous melanoma, cutaneous squamous cell carcinoma (CSCC), skin lymphomas, cutaneous lymphoma, and Merkel cell carcinoma (MCC). In this type of disease, miRNAs are described as potential biomarkers to diagnose early lesion and/or early metastatic disease. In the future, whether in tissue or circulating in body fluids, miRNAs will gain their place in skin cancer diagnosis, prognosis, and future therapeutic targets.

## Introduction

In the last decade, the dramatic increase of diseases that are linked to changes in RNA modifications has shown that the epitranscriptomic domain will impact health science. Innovative methods used for molecular modification pattern detection will further lead to the expansion of improved diagnosis and new therapeutics will be approved for various human diseases (Jantsch et al., 2018).

MicroRNAs (miRNAs) are a large group of short, non-coding RNA molecules that are approximately 22-nt long and are involved in post-transcriptional control of gene expression (Quinodoz and Guttman, 2014). Their action is completed by base pairing with messenger RNA (mRNA) transcripts that encompass target sequences, resulting in an increase of mRNA decay and/or translational attenuation (Caron et al., 2012). Through this action, miRNAs are involved in many physiological processes, and any deregulations at this level will trigger abnormalities and further human diseases (Cretoiu et al., 2016a, b). The involvement of miRNAs in pathological processes has made them to be recognized as potential therapeutic targets as well as future biomarkers with diagnostic and/or prognostic potential (Horsham et al., 2015; Monroig-Bosque Pdel et al., 2015).

There is also a series of unconventional roles for these molecules. Thus, miRNAs can activate Toll-like receptors displaying a pro-inflammatory/pro-metastatic potential, and hence, specific miRNAs can become future therapy targets. Also, miRNAs can act on protein expression in a cell cycle-dependent manner. For example, miR-369 directs the association of Argonaute (AGO2) protein and fragile X mental retardation-related protein 1 (FXR1) with AU-rich elements (AREs) in tumor necrosis factor alpha (TNF-a) mRNA to activate translation (Vasudevan et al., 2007). Nevertheless, miRNAs associate with non-AGO proteins acting as a decoy for another RNA binding protein interfering with its function. At the mitochondrial level, miRNAs have been shown to inhibit mitochondrially encoded cytochrome c oxidase subunit 1 (MT-COX1) protein expression while increasing MT-COX2 mRNA expression (Das et al., 2012). miRNAs that are localized in the nucleus can inhibit the maturation of other miRNAs *via* direct interaction with the primary transcript, an interaction that can lead to hindering apoptosis. Last, but not least, miRNAs can directly activate transcription. Therefore, miRNAs with AGO1/AGO2 can be imported into the nucleus and bind to the promoter RNA of cyclooxygenase-2 (COX2), leading to COX2 transcription (Matsui et al., 2013). Classic miRNAs functioning along with unconventional pathways prove that miRNAs are involved in complex cellular regulatory functions (Dragomir et al., 2018).

Therefore, miRNAs were brought into the spotlight because they were found to regulate and to be regulated in tumorigenesis seminal processes like tumor development, progression, and metastasis/aggressiveness of all types of cancer (Albulescu et al., 2011; Bobbili et al., 2017). For example, recently, the miR-17-92 cluster was identified as overexpressed in many tumors, promoting uncontrolled cell proliferation (Liu et al., 2017; Zhang et al., 2018). Within this cluster, miR-17-5p is associated with cancer aggressiveness and therapy responsiveness in liver, gastric, or colorectal cancers, where it has an oncogene function. In other cancers, such as breast, prostate, and lung cancers, it can have a tumor-suppressive action (Li et al., 2017; Liu et al., 2017). Moreover, miR-17-5p has also been found elevated in the circulation of patients diagnosed with various cancers (Monroig-Bosque Pdel et al., 2015; Bobbili et al., 2017). Another example is miR-7, a molecule generated from three different genes, regulating major cellular processes, this finding pointing out the complexity of miRNA generation. Besides several other cancers, miR-7 is involved in skin cancer, having the potency to be further developed as a biomarker and future therapy target (Horsham et al., 2015).

Mouse models are used to decipher the dynamics of molecular events. In a skin carcinogenesis mouse model, the miRNA-200 family members were found correlated with staging and progression. miR-205-5p overexpression in spindle cancer cells was shown to decrease tumor cell proliferation and invasiveness (Skourti et al., 2016).

Out of all skin cancers, the most abundant studies regarding miRNA evaluation are developed in cutaneous melanoma.

In this direction, miR-214, a pleiotropic molecule, was found deregulated in melanoma, this RNA molecule coordinating important signaling networks (e.g. PTEN/AKT, β-catenin, and tyrosine kinase receptor pathways), gene expression modulators (e.g. Ezh2, p53, and TFAP2), and even other miRNAs like miR-148b. Through all these functions, it is involved in tumor cell proliferation, in particular tumor cell characteristics like stemness, invasiveness, and other complex processes like angiogenesis and metastasis (Penna et al., 2013). Once more, this shows that this miRNA can be a potential diagnostic/prognostic biomarker in skin cancer, this finding pointing out that there are miRNAs that have a ubiquitous role in tumorigenesis (Penna et al., 2015). Almost concomitantly, another group has shown that skin cancer is associated with the methylation status of miRNA-148a. Using methylation-specific PCR, it was demonstrated that, in tumor tissues, DNA methylation of miR-148a was higher compared to healthy tissues. Moreover, miR-148a methylation status was correlated with various parameters (e.g. age, pathological differentiation, and lymph node metastasis) and with patient’s survival; therefore, miR-148a methylation status can be a candidate for a prognostic biomarker in skin cancer (Tian Y. et al., 2015).

In the last decade, several other miRNAs were associated with skin cancers, as further described in the following sections. One important note from the epitranscriptomic domain is that these miRNAs can have dual functions: pro- or anti-tumoral action.

Nowadays, miRNA expression uses microarrays, bioinformatics analysis, and finally validation with qPCR. In the future, complex technologies are to be used to identify miRNA molecules that can identify particular subgroups of patients with worse prognosis.

## Main Technologies That are Used for miRNA Identification From Biological Samples

Aberrant miRNA expression was found in tumors and biological fluids. More and more data show that miRNAs could be excreted into circulation *via* extracellular vesicles or bound to proteins such as AGO or high-density lipoprotein (HDL) (Larrea et al., 2016; Voichitoiu et al., 2019). Thus, circulating miRNAs may serve as a reflection of the underlying disease, but this approach is hampered for the moment by the complicated and lengthy PCR-based procedures used by most laboratories.

MicroRNAs as non-coding RNAs (ncRNAs) are transcribed by RNA polymerases (Pol) II and III and, upon transcription, generate primary transcripts (pri-miRNAs). These pri-miRNAs can comprise one or more miRNAs, and from the structural point, these molecules are 5′-capped and polyadenylated (Hube and Francastel, 2015). Pol III also transcribes a minor group of miRNAs associated with Alu repeats (Lin and Gregory, 2015; Taucher et al., 2016). pri-miRNAs are further processed in the nucleus by a complex composed of two proteins: the double-stranded RNA-specific RNase DROSHA and the RNA-binding protein DGCR8 (DiGeorge Syndrome Critical Region 8). Upon processing, a precursor miRNA (pre-miRNA) is generated, which is afterward cleaved into a mature miRNA by DICER, an RNAse III enzyme (Kozomara and Griffiths-Jones, 2011). From the technological point of view, this transcription molecular flow can be depicted using several high-end approaches. Moreover, these applied methodologies are used to establish whether the studied miRNA(s) have a pro- or anti-oncogenic role in a certain cellular process, in both time and space. When a gene that encodes an oncogenic miRNA is overexpressed, amplified, or its epigenetic silencing is hindered, an anti-oncogenic pathway is deregulated. Dissimilarly, if deletion, mutations, or epigenetic silencing is active on a tumor-suppressive miRNA that would normally regulate oncogenes, this may lead to enhanced oncogenic activity (Markopoulos et al., 2017).

Technologies are diverse and have specific characteristics; thus, if some of them analyze many targets in a few samples (like sequencing and microarrays), others analyze few targets in many samples (like quantitative reverse transcription PCR, qRT-PCR).

### Quantitative PCR Methods

The quantitative PCR (qPCR) technique struggles between perception and reality. While it is perceived as a precise and quantitative data reflecting the tested experimental parameters, without strict guidelines, validation, and data analysis procedures, the obtained results can be frequently false and opposed to the actual process developed in the experiment (Sanders et al., 2018). The reality is that the planning process is fundamental for the correctness of the results, and protocols [sample handling, harvesting, nucleic acid extraction, reverse transcription (RT), and qPCR] should be carefully designed to obtain the correct results.

Along with microarray platforms, quantitative real-time PCR (RT-qPCR) technology is used for investigating tissues or circulating miRNAs, especially in the cancer diagnosis biomarker field. The two most powerful methods used in this endeavor are relative quantification PCR and absolute qPCR. The major difference between these two is related to the suitable internal/external controls in PCR reaction. qPCR overpasses this issue, but has other technical difficulties related to the standard curve needed for specific miRNAs. This issue claims a series of standards, making the method laborious and expensive (Wang and Chen, 2014). The RT-PCR methodology is developed in several versions such as TaqMan, two-tailed RT-qPCR miRNA, miRCURY LNA qPCR assay, and so on, depending on the actual manufacturing company (Androvic et al., 2017; Leti and DiStefano, 2018).

The RT-PCR principle comprises the first step of complementary DNA (cDNA) synthesis, followed by the detection of amplified products checked by a conventional PCR version. The RT-PCR method detects short-length miRNA targets; hence, the miRNA primers for both cDNA synthesis and detection must be carefully designed to assure the RT-qPCR’s specificity and sensitivity. This step is followed by the absolute quantification of small miRNA panels. Although it is easy to be included into routine measurement workflow with robust automation, RT-PCR cannot be enrolled for new miRNA species detection; moreover, the reaction conditions may differ according to each miRNA due to sequence-specific differences in primer annealing (Dave et al., 2019). Specific miRNAs are altered in solid tumors, including melanoma, and hence, RT-PCR is very useful in detecting such modified expressions.

Normalization strategy is a critical point in this technology and accounts for the variability of results. Classically, “housekeeping” genes are used for the normalization of qPCR data and are actually endogenous controls subjected to the same experimental workflow as the target genes. While quantifying miRNAs, the references used are stable small RNA controls, typically small non-coding RNAs, small nuclear RNAs, and small nucleolar RNAs (e.g. SNORD44, SNORD48, and RNU6-1). When quantifying circulating miRNAs, normalization cannot be done with the above-mentioned small RNAs because they are variable (Reid et al., 2011; Benz et al., 2013). In this case, an exogenous synthetic RNA spike-in control for normalization could be used, for example, *Caenorhabditis elegans* cel-miR-39 or *Arabidopsis thaliana* ath-miR-159a. This external reference, although subjected to the same workflow, will not overcome the sources of variability, like total circulating miRNA concentration that is inter-individually and/or is disease-associated (Marabita et al., 2016).

Quantitative RT PCR analysis was used by Wang and Zhang (2015) to characterize the expression levels of miR-203 in 148 cases of melanoma tissues and adjacent non-cancerous tissues. They demonstrated that miR-203 expression was significantly decreased in melanoma tissues, and its downregulation was significantly associated with tumor thickness and tumor stage.

In a recent study, miR-29a’s role in repressing melanoma was highlighted. Thus, RT-PCR was used in a complex molecular approach for demonstrating the downregulation of miR-29 in an A375 melanoma cell line; miR-29a could potentially suppress melanoma by negatively regulating apoptosis-related protein Bmi1 (Xiong et al., 2018). The intra-tumor expression of miRNA could be a biomarker for predicting melanoma patients’ survival. Thus, in a recent study using RT by means of the TaqMan version for miRNA RT on over 130 primary and metastatic tumors, several miRNA expressions were shown. Especially for primary melanomas, this approach showed the downregulation of intra-tumor expression for several miRNA species, such as miR-125b, miR-182, miR-200c, and miR-205, which could promote tumor dissemination. The TaqMan method could label miRNA-125b, miRNA-200c, and miRNA-205 as useful prognostic biomarkers correlated with shorter survival and, thus, able to select high-risk patients (Sanchez-Sendra et al., 2018). A notable version of the TaqMan method for miRNA detection is *SplintR-qPCR*, the major difference between the SplintR-qPCR and TaqMan being related to the tactics used for cDNA synthesis. While the SplintR involves a ligation step of complementary oligonucleotides followed by qPCR to generate a DNA copy of the miRNA, the TaqMan relies on a miRNA-specific DNA hairpin as a primer for newly created cDNAs (Jin et al., 2016). Regardless of the technique version enrolled, RT-PCR remains, for the moment, the gold standard for cancer research and for diagnostic purposes (Hunt et al., 2015).

### Microarray Platforms

The microarray principle relies on target hybridization with a specific probe usually bound on a solid surface to measure the miRNA/DNA abundance based on the fluorescence detection method (Wang and Xi, 2013). This technology is usually applied in characterizing previously identified miRNA species. The main advantages of the microarray assay rely on approaching hundreds of miRNA expression targets, high throughput, and speed of detection, providing mostly a relative quantification of miRNA. In cutaneous melanoma, these types of microarray platforms are mainly used to identify circulating miRNAs as diagnostic markers. One of the first attempts in this line was completed by Leidinger et al. (2010) by detecting a panel of 16 species of miRNAs isolated from a patient’s blood. These miRNAs can discriminate with high sensitivity and specificity between melanoma metastatic cases (stages III and IV) and healthy subjects. Later on, Komina et al. (2016), using a microarray and, subsequently, validation by RT-PCR, demonstrated that the expression of over 1,100 miRNAs was altered in melanoma samples compared to melanocytic nevi. Moreover, they showed that miR-4286 mediates the proliferation and apoptosis in melanoma cells, having a pleiotropic effect by triggering several pathways (Komina et al., 2016).

A more recent study by Van Laar et al.’s (2018) group performed microarray profiling on the plasma of patients with stages I–IV melanoma and compared the profiles with those obtained in healthy subjects. The study discovered a 38-miRNA panel able to differentiate between melanoma and normal controls with high sensitivity and specificity (Mumford et al., 2018).

However, apart from the sensitivity and specificity of the detection, the necessary microarray equipment is fairly expensive as well as the required personnel expertise, and therefore, microarray platforms are rather suitable for miRNA expression surveys concerning a certain disease (Krepelkova et al., 2019; Ouyang et al., 2019).

### miRNA Sequencing

Technologies like qPCR and microarrays are viewed as traditional techniques for miRNA expression because qPCR is a sensitive technique, can evaluate a dynamic molecule range, and its workflow can be accessible for any lab. However, this technology has its intrinsic limitations, as described in the section “Quantitative PCR Methods.”

Next-generation sequencing (NGS) for miRNAs (miRNA-seq) comes with a series of key advantages (Ozsolak and Milos, 2011). Therefore, the sequence of a certain miRNA can be unknown, so the technology can be used in the discovery phase. It has an improved specificity for members of the same miRNA family, where the technique can distinguish molecules only one to two bases different. However, the main limitation of this technology is the computational platform that allows data interpretation. Various software programs have been developed, and most recently, a comprehensive pipeline analysis, *miARma-Seq*, was published, identifying mRNAs, miRNAs, and circular RNAs (circRNAs), along with evaluating differential expression, target identification, and functional evaluation (Andres-Leon et al., 2016, 2018). Recent data, as detailed further, have shown that, using this technology for miRNA discovery in melanoma, new miRNA panels were identified associated with clinical patient prognosis (Jayawardana et al., 2016).

### miRNA Enzyme Immunoassay

miRNA enzyme immunoassay (miREIA) represents a relatively novel immunoassay technology designated for miRNA measurement in biological samples. Briefly, miRNAs isolated from a biological sample (e.g. blood) are hybridized to a biotinylated oligonucleotide, resulting in DNA/RNA hetero-hybrids that further would be incubated with streptavidin-labeled microparticles. Next, an antibody labeled with acridinium ester would specifically bind to the resulted hybrids quantitatively, proportional to the hetero-hybrid amount and, therefore, to the amount of the specific miRNA from the analyzed sample. The coupling reaction is identified by chemiluminescence-based detection (Kappel et al., 2015). The entire workflow for miREIA is analogous to ELISA, which is suitable for biomedical purposes and is routinely used in clinical laboratories. Therefore, this novel miRNA measurement approach would be soon extended to novel cancer types and new detected miRNA species that will be linked with the tumorigenesis of human cancers, including skin melanoma (Kappel et al., 2015; Thyagarajan et al., 2019). Using this method, it was shown that miR-150-5p is upregulated in patients of all stages of melanoma and that the diagnostic sensitivity and specificity were greatly improved when this miRNA was included along with miR-149-3p and miR-193a-3p (Mumford et al., 2018). Future results will be obtained with the newly available miREIA kit hsa-miR-150-5p.

### miRNA Multiplexing Systems

Multiplexed miRNA analysis comprises the use of the flow cytometry principle and PCR. The detection involves a three-dimensional hydrogel particle (Tanase et al., 2011) that can sustain good hybridization properties and large nucleic acid binding capacity. The method can analyze plasma, serum, exosomes, saliva, and urine, but it can also be used on tumor tissue and/or tumor cell suspensions. It can detect a range of 5–400 miRNAs from a single sample. The principle behind this technology is very simple: each particle type has a barcode that is specific for the analyzed miRNA; when the biological sample is in contact with these particles, the target miRNA will bind to its specific particle. This first step is then followed by molecular steps that will lead on each particle to a bound DNA–miRNA–DNA complex. This complex is amplified through PCR using specific oligos and, adding, in the end, a biotin label to the PCR product. Like in classical flow cytometry, the biotin is labeled with a streptavidin reporter complex that will be read in a cytometer. The fluorescent intensities of each coded particle will indicate the miRNA expression level (Elias et al., 2017; Leng et al., 2018). Multiplexed microarray profiling was used to identify let-7b and miR-199a as the most significant discriminators associated with metastasis in uveal melanoma, and the results were validated by qPCR (Worley et al., 2008).

Another multiplex-based assay is one that uses Firefly technology (FirePlex^®^ miRNA Assay V2 – Assay Protocol, Protocol Booklet version 2.2, February 2019), and this assay is based on FirePlex^®^ particles. These particles are bioinert hydrogels that are coupled with the post-hybridization labeling method. They have a site for a specific miRNA and another one for tagging, so that the actual binding will be detected in this type of multiplexing *via* fluorescence.

This technology was used for detecting miRNAs in brain diseases (Hoss et al., 2015), in brain metastases of breast cancer (Le et al., 2014), or, more recently, in Richter syndrome, the syndrome that shows the switch of chronic lymphocytic leukemia into its aggressive form (Van Roosbroeck et al., 2019). Unfortunately, although this technology has good potential, reports using this assay in skin cancer are not yet published.

**FIGURE 1:**
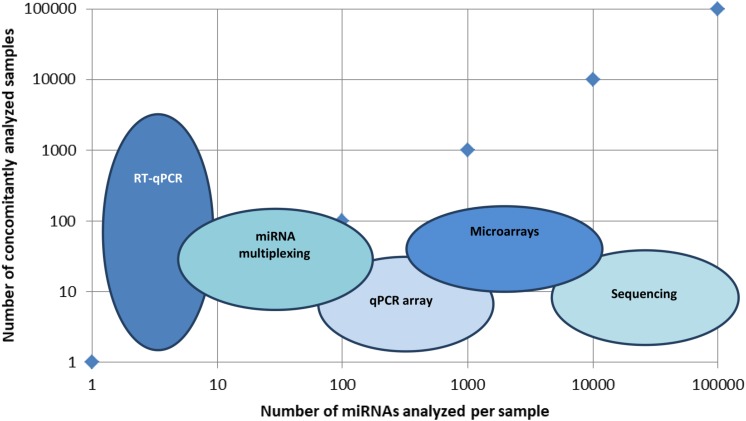
Main technologies used in miRNA identification. Graphical representation of several miRNAs identified per sample *versus* a number of concomitantly analyzed samples. Technologies like RT-qPCR identify a few types of miRNAs, but in a large set of samples, miRNA multiplexing can identify hundreds of miRNAs in hundreds of different samples, while microarrays and sequencing can identify up to hundreds of thousands of miRNAs in hundreds of different samples.

There is a large panel of technologies that aid miRNA detection, and one of the main issues is to make the proper selection for the flow of technologies that will be providing high throughput and validation of the searched miRNA molecules. In Figure 1, a visual representation of the main types of technologies put in use in miRNA detection in terms of high-throughput detection in multiple samples is presented.

### Sampling for miRNA Detection

As presented in the previous section, assays that identify miRNAs in tissue and/or in circulation are very diverse.

Tests that focus on circulating miRNAs can have several advantages in comparison to a tissue biopsy, which is an invasive procedure (Young et al., 2007), Moreover, if the tumor is small, there is an increased failure of the biopsy, this case being frequent in small skin tumors as in other types of cancers (Corcoran et al., 2012). Therefore, in terms of availability and the invasiveness of the procedure, circulating miRNAs are the preferred test. As presented in several sections, circulating miRNA have a superior robustness and stability against tissue miRNAs (Mitchell et al., 2008). For monitoring the therapy and overall clinical outcome of a patient, it is more proper to use miRNAs that circulate in whole blood, plasma, serum, and urine (Lawrie et al., 2008). Important challenges in using miRNAs as biomarkers in oncology still remain. Therefore, there are conflicting results that show a miRNA as an oncomir in one type of cancer while in others the same molecule is a tumor suppressor. Probably all these results come from using different technologies, different methods to enrich and identify a certain miRNA or a panel of miRNAs, and, last but not least, the sample type, tissue, or plasma (Git et al., 2010; de Planell-Saguer and Rodicio, 2011).

Circulating miRNAs are generated from tumor cells, circulating normal/abnormal cells, or even other non-tumor tissues; therefore, the picture of circulating miRNAs has a wholesome characteristic (Philippidou et al., 2010; Greenberg et al., 2011).

In both circulation and tissue, miRNA identification stumbles upon an important issue, namely, having proper control of the miRNA expression (Greenberg et al., 2011). If tissue sampling can partially overcome this issue when compared to normal tissue, in circulation this still remains a point to be argued for.

Some of the advantages and disadvantages of evaluating miRNAs in tissue samples *versus* body fluid samples are resumed in Table 1.

**TABLE 1:** Sample type advantages and disadvantages for miRNA identification.

**Sample**	**Advantages**	**Disadvantages**
Serum/plasma/urine	miRNAs are stable and robust. Non-invasive approach, easily repeated. Offers real-time monitoring of the disease, of the therapy, of the clinical outcome of the patient. Can be applied for early detection.	Detection methods are various, usually non-standardized, and with low sensitivity. The selected panel of miRNA can have non-detectable levels. Circulating miRNAs can have various origins besides tumor cells *per se*: immune cells, platelets, and so on, thus an increased variability.
Tissue	Methods are usually standardized having high sensitivity. miRNAs are disease-related.	Invasive, difficult to repeat. Does not detect the initiation of metastasis at distant organs. It is not proper for therapy monitoring.

## miRNAs Detected in Cutaneous Melanoma

The majority of publications regarding miRNA evaluation focus on cutaneous melanoma. The reasons for this concentrated research rely on the highly metastatic potential of this skin cancer, increased recurrence, resistance to standard chemotherapies, and the high genomic heterogeneity. Thus, several areas study miRNAs in melanoma: in the accurate differentiation between other skin cancers, in developing new therapeutic targets, and in evaluating the prognostic and diagnostic potential of these molecules.

Cutaneous melanoma has a particular molecular biology due to the many genetic alterations that have been identified, and this feature has triggered the development of targeted therapies. But not only genetic alterations, which characterize this skin cancer, epigenetic deregulation was also identified concerning melanoma initiation and progression (Luo et al., 2014). Deregulated miRNA expression was identified in miRNAs involved in the tumor cell cycle, proliferation, migration, invasion, apoptosis, and the triggered immune response. Abnormal hypermethylation was identified in over 70 genes that are involved in cutaneous melanoma pathogenesis (de Unamuno et al., 2015), and alterations identified in the miRNA-processing enzyme DICER were reported several years ago. Besides the involvement in seminal melanoma tumorigenesis, miRNAs interact with the main transcription factor of melanocyte, a microphthalmia-associated transcription factor (Leibowitz-Amit et al., 2012). It was reported that over 800 different miRNAs can be found within cells and that their expression will vary during tumorigenesis stages. In melanoma, miR-21, miR-125b, miR-150, miR-155, miR-205, and miR-211 were the molecules that were searched for their prognostic value and for which targeted therapy was developed to obliviate their onco-miRNA action (Latchana et al., 2016). Relating these miRNAs with the main molecular deregulated pathways, e.g. the RAS/MAPK pathway, the MITF pathway, the p16INK4A-CDK4-RB pathway, and the PI3K-AKT pathway, would offer new insights in deciphering the aggressive and metastatic feature of melanoma (Segura et al., 2012; Greenberg et al., 2014).

In 2019, a complex retrospective study based on mRNA-seq, miRNA-seq, and DNA methylation was performed on tissue samples of almost 450 patients. In the group that had the best prognosis, immune-related genes were found upregulated, along with DNA hypomethylation and a high number of mutations. The mutational signature in the better prognosis group was associated with ultraviolet light exposure. This integrated study highlighted once more the molecular heterogeneity of cutaneous melanoma (Li and Cai, 2019).

Several years ago, when evaluating stage III melanoma patients, it was shown that the standard clinical/pathological information needs to be combined with information developed by various omics platforms (Jayawardana et al., 2015). It becomes more and more obvious that information coming from the proteomic domain must collide with the information from the genomic, transcriptomic, and metabolomic domains (Neagu et al., 2019b) to provide an individualized treatment approach for the patient.

### miRNAs as Diagnostic and Prognostic Markers in Cutaneous Melanoma

In the search for their diagnostic and prognostic potential, extensive work was done in evaluating miRNA expression in tumor tissue, isolated tumor cells, tumor microenvironment, and body fluids like serum/plasma. In comparison to tissue-related studies, reports regarding the circulating level of miRNAs in melanoma patients are scarce.

#### Circulating miRNAs in Cutaneous Melanoma

MicroRNAs can circulate in the blood completely free or complexed with proteins, or in vesicles, like exosomes and microvesicles. As already mentioned, these circulating forms are stable, but a disadvantage in terms of specificity is that they can have different origins. They can originate from the primary tumor, but also from a tumor metastasis, being the result of cancer cells or even of other cells from the tumor microenvironment. Then, circulating mRNAs can originate from inflammatory circulating cells, immune cells, and any cells that are involved in an anti- or pro-tumor response (Taylor and Gercel-Taylor, 2013; Ma et al., 2014). Taking into account these bottlenecks (see also Table 2), studies regarding the identification of circulating mRNAs in melanoma patients started >10 years ago.

**TABLE 2:** Overview of the main miRNAs found in melanoma tissue or/and in circulation in patients diagnosed with cutaneous melanoma.

**miRNAs**	**Sample**		**Observation**	**References**
	**Serum/plasma**	**Tissue**		
miR-214		X	Regulates PTEN/AKT, β-catenin, tyrosine kinase receptor pathways, genes *Ezh2*, *p53*, *TFAP2*, and other miRNAs like miR-148b	Penna et al., 2015
miR-148a		X	Downregulated; is regulated by DNA methylation; an independent indicator of prognosis	Tian Y. et al., 2015
miR-221	X	X	Downmodulates p27Kip1/CDKN1B and c-KIT receptor; favors tumorigenesis	Kanemaru et al., 2011; Friedman et al., 2012
miR-16	X		Correlates with tumor thickness, ulceration, stage, and tissue Ki-67 expression	Guo et al., 2016
miR-29c		X	Downregulated and correlates with stage, overall survival; regulates cell surface glycoprotein B7-H3	Nguyen et al., 2011; Wang et al., 2013
miR-146a-5p		X	Regulates Toll-like receptor, NF-κB, ErB, and measles signaling pathways; regulates 38 target genes; most important NRAS gene	Aksenenko et al., 2019
miR-205		X	Low expression correlates with shorter survival	Hanna et al., 2012
Pattern miR-142-5p, miR-150-5p, miR-342-3p, miR-155-5p, miR-146b-5p		X	Associated with patient clinical prognosis	Jayawardana et al., 2016
miR-10b		X	Increased expression in primary melanomas and further increased in metastases	Saldanha et al., 2016
miR-203		X	Decreased expression in tissues compared to healthy tissue; associated with tumor thickness and stage	Wang and Zhang, 2015
let-7a and let-7b, miR-148, miR-155, miR-182, miR-200c, miR-211, miR-214, miR-221, miRNA-222	X	X	Linked to NRAS, microphthalmia-associated transcription factor, receptor tyrosine kinase c-KIT, and AP-2 transcription factor	Mirzaei et al., 2016
miR-106b		X	High expression correlated with Breslow index, ulceration, and clinical stage	Lin et al., 2015
7 miRNAs (MELmiR-7)	X	X	MELmiR-7′ correlates with overall survival	Stark et al., 2015

Thus, work done almost a decade ago has shown that miR-221, which is aberrantly expressed in melanoma cells, can also be found in the sera of patients diagnosed with cutaneous melanoma. This miR-221 downmodulates p27Kip1/CDKN1B and the c-KIT receptor in melanocytes and, hence, favors tumorigenesis. Using quantitative real-time polymerase chain reaction, circulating miR-221 was quantified in a patient’s serum and was found significantly higher compared to controls. Moreover, the serum level correlated with stage, the tumor thickness, and recurrence. It is one of the first published reports that show a circulating miRNA that has good potential as a prognosticator in melanoma (Kanemaru et al., 2011). Later on, serum miR-221 was investigated by another group as well using RT-qPCR. Circulating miR-221 was also found significantly increased in patients’ sera and identified as an independent prognosticator of worse outcome, reinforcing the previously published data (Li et al., 2014).

Another group has investigated 355 miRNAs in the sera of melanoma patients using qRT-PCR technology. Out of all these screened circulating miRNAs, a pattern of three miRNAs could classify high- *versus* low-recurrence-risk patient groups and was associated with tumor burden. This was one of the first studies to identify circulating miRNAs in melanoma, and this finding would indicate the prognostic value of circulating miR-199a-5p, miR-33a, and miR-424 (Friedman et al., 2012).

While some of the circulating miRNAs are found elevated, there are also studies showing decreased miRNA circulating levels. Thus, in a patient’s serum, a significant reduction of the circulating miR-16 level was reported when compared to controls. Serum miR-16 correlated with tumor thickness, ulceration, stage, and tissue Ki-67 expression. This miRNA also has prognostic value as it independently predicted patients’ survival outcome (Guo et al., 2016). miR-16 was previously included in the miRNA panel reported by another group (Stark et al., 2015).

Using RT-qPCR, circulating miR-206 was detected in low concentrations compared to controls. Moreover, low serum miR-206 indicated patients with melanoma metastasis and a significantly shorter overall survival. This finding indicated circulating miR-206 as a possible prognostic biomarker (Tian R. et al., 2015).

Blood-based molecules with a biomarker role can improve early detection of metastasis, therapy initiation, and, hence, patient prognosis. In this picture, the recently developed “liquid biopsy” using blood samples gains more and more utility in melanoma disease monitoring. From blood samples, circulating tumor cells (CTCs) can be detected along with cell-free circulating tumor DNA (ctDNA) and circulating miRNAs (cmiRNA). These tests performed some years ago on stages III and IV melanoma patients have shown that these molecular combined analyses (CTC, ctDNA, and cmiRNA) can lead to individualized therapy. In this light, technologies like massive parallel sequencing (MPS) can aid the classical molecular assays to enlarge the panel of powerful biomarkers (Huang and Hoon, 2016).

Circulating free miRNAs populate the serum because they are released into the interstitial fluid and, further, in the main circulation. They are stable even outside the cell(s), can be tissue-specific, can vary with cancer dynamics, and change according to disease progression or therapeutic response. All these characteristics make them good blood-based biomarkers. The downfall of these molecular biomarkers consists in the analytical methods, in the normalization strategies that need standardization before entering clinical tests (Polini et al., 2019).

Panels of circulating miRNAs were also studied. Aberrant genes associated with melanoma (e.g. NRAS, microphthalmia-associated transcription factor, receptor tyrosine kinase c-KIT, and AP-2 transcription factor) were found linked to the aberrant activation of sets of circulating miRNAs (e.g. let-7a and b, miR-148, miR-155, miR-182, miR-200c, miR-211, miR-214, miR-221, and miR-222) and can aid the large set of potential biomarkers and/or therapeutic targets in melanoma (Mirzaei et al., 2016).

In a combined serum and tissue analysis, it was reported that the expression of a set of 17 miRNAs (MELmiR-17) can select some good prognosticators. In serum, a seven-miRNA set (MELmiR-7) characterized the overall survival of patients even better than did the classical serum markers lactate dehydrogenase (LDH) and S100B. This MELmiR-7 could indicate melanoma progression, recurrence, and survival to detect any very early relapse. MELmiR-7 consists of miR-16, miR-211-5p, miR-4487, miR-4706, miR-4731, and miR-509-5p (Stark et al., 2015).

#### Tumor Tissue miRNA Expression in Cutaneous Melanoma

Tissue analysis of miRNA expression goes back more than 10 years ago when tissue miR-221 and miR-222 were reported as abnormally expressed. These miRNAs were found downregulating c-KIT receptor and p27Kip. This finding highlighted that a new therapeutic pathway can be opened using the regulatory potential of miRNAs (Felicetti et al., 2008). It is to be noted that miR-221 was also later found in circulation in melanoma patients (Kanemaru et al., 2011).

Using quantitative *in situ* hybridization (qISH) on over 100 primary melanomas, a test that was further validated on over 200 additional samples, low levels of miR-205 were shown to be correlated with lower survival in patients. Moreover, these low levels were independent of stage, age, gender, and Breslow index. Thus, miR-205 was reported as a tumor suppressor miRNA in melanoma (Hanna et al., 2012).

Using RT-qPCR, several isoforms of miR-29 were studied. Out of all the studied isoforms, reduced expression of miR-29c was correlated with late stages of melanoma. Moreover, the hypermethylation status of the promoter region of tumor-related genes (TRGs) and non-coding MINT loci is in opposite correlation with miR-29c expression. MiR-29 downregulates DNMT3A and DNMT3B (DNA methyltransferases) that methylate TRGs. DNMT3B expression and miR-29c were found significantly correlated with the overall survival of patients. These molecular traits can be epigenetic biomarkers in melanoma prognosis (Nguyen et al., 2011). MiR-29c regulates a cell surface glycoprotein, B7-H3, namely its down-expression. Using RT and RT-PCR, it was shown that B7-H3 is overexpressed and, furthermore, that it is correlated with the migration and invasion of melanoma cells (Wang et al., 2013).

Using RT-qPCR, miR-10b was shown increased in primary melanoma tissues, and further, its levels were found increased in melanoma metastasis. The combination of miR-10b and miR-200b showed that their expressions have independent prognostic value. Using identification of these miRNAs on aggressive thick melanomas (Saldanha et al., 2016), miR-203 expression was found to be significantly decreased in melanoma tissues, while its downregulation was found to be associated with tumor thickness, stage, and reduced overall survival rate (Wang and Zhang, 2015).

As previously established, an inverse correlation between tyrosinase-related protein 1 (TYRP1) mRNA expression in metastatic tissues and survival was reported. An additional study of miR-155 involvement was reported several years ago. TYRP1 mRNA has two miR-155-5p binding sites. In melanoma cell lines, it was demonstrated that miR-155 induced TYRP1 mRNA decay and, in metastatic tissues, TYRP1 mRNA inversely correlated with miR-155 expression. This mechanistic study proved that polymorphisms in the 3′-UTR of TYRP1 mRNA affect the regulation performed by miR-155 and, further, its translation into the protein. Therefore, there are subgroups of melanoma patients that display this polymorphism, hindering miR-155 regulation and proving its prognostic power (El Hajj et al., 2015). An additional study published in 2019 has shown that miR-155 has a new target, namely, protein kinase WEE1. In an experimental mouse model, it was shown that miR-155 increased the expression and silenced WEE1, leading to decreased metastases. In the late stages of melanoma, during the metastasis process, decreased miR-155 and increased expression of WEE1 significantly contributed to the metastatic potential of melanoma cells (DiSano et al., 2019).

Another miRNA that was studied in melanoma tissues and in tumor cell lines is miR-675, whose expression was found downregulated. Using functional assays, it was demonstrated that in upregulating miR-675, hindrance of tumor cell proliferation and invasion was obtained. In the search for its target and using an array of technologies (e.g. bioinformatics analysis, luciferase reporter assay, RT quantitative polymerase chain reaction, and Western blot analysis), it was demonstrated that metatherian (MTDH) is the direct target of miR-675; therefore, miR-675 can be further developed as a potential therapeutic target (Liu et al., 2018). Recently, in melanoma cell lines and further in tissues, miR-135b expression was studied and found overexpressed. Using luciferase reporter assay and Western blot analysis, the target gene for this miRNA was found to be the large tumor suppressor kinase 2 (LATS2). In melanoma cell lines, if this miRNA was found to be overexpressed, cell proliferation and migration was enhanced; conversely, its low expression suppressed growth/metastasis and increased tumor cell apoptosis. This molecular tandem miR-135 and LATS2 can decipher new oncogenic mechanisms in melanoma (Hu et al., 2019). Another recent finding showed miR-204 as a complex regulator in melanoma. Using a large panel of methods [e.g. Western blotting, qRT-PCR, and chromatin immunoprecipitation (ChIP) assay], it was demonstrated that Semaphorin-5A (Sema5A) regulates cell migration and invasion. Sema5A expression was controlled by the transcription factor c-Myb, and if miR-204 overexpression was induced in melanoma cell lines, a concomitant decrease of Sema5A, Bcl-2, and c-Myb protein expression was observed (D’Aguanno et al., 2018).

Panels of tissue miRNAs were also the focus of recent studies. In tumor tissue, the downregulation of miR-125b, miR-182, miR-200c, and miR-205 expressions was reported from primary melanomas to metastatic samples. The combination of miR-125b, miR-200c, and miR-205 was found to be correlated with shorter survival. The authors highlight that the downregulation of miR-205 alters the melanoma cells’ interaction with the extracellular matrix favoring metastasis. The panel of miR-125b, miR-200c, and miR-205 can be developed in a prognostic biomarker panel and for selecting high-risk-recurrence patients (Sanchez-Sendra et al., 2018).

Earlier studies have shown that global miRNA expression profiles are differentially expressed in correlation with *BRAF* mutation. Therefore, when *BRAF* mutation appears, several miRNAs were found underexpressed, namely, miR-193a, miR-338, and miR-565. The association of low miR-191 expression with high miR-193b expression is associated with poor survival. Authors pointed out that a miRNA classifier is needed to validate prognostic miRNA biomarkers (Caramuta et al., 2010). In the same year (2010), using miRNA arrays, 18 miRNAs were identified as associated with survival. Out of this set, a six-miRNA signature could prognosticate stage III patients’ clinical evolution. This panel of miRNAs comprised the following molecules: miR-150, miR-342-3p, miR-455-3p, miR-145, miR-155, and miR-497 (Segura et al., 2010).

Evaluating in a meta-analysis a large set of 16 studies that identified 25 miRNA expressions in over 2,600 melanoma patients, the association between survival outcome and miRNA expression was analyzed. A subgroup emerged where miR-10b, miR-16, and miR-21 were associated with poor prognosis. This study, published in 2018, showed the need to largely analyze the prognostic power of miRNAs (Sabarimurugan et al., 2018).

#### Tumor Microenvironment miRNA Expression in Cutaneous Melanoma

Like in other solid tumors, the melanoma tumor microenvironment has an important role in the tumorigenesis dynamics (Neagu et al., 2019a). The tumor microenvironment consists of cancer-associated fibroblasts (CAFs), tumor-associated macrophages (TAMs), endothelial cells, immune cells, and a myriad of molecules with different origins. From this amazing concert of molecules in the tumor microenvironment, miRNAs have a paracrine function. Melanoma tissue and the peritumoral region were subjected to microarray analysis using Gene Atlas Microarray System, further validated by RT-PCR. This study, published in 2019, has revealed over 140 different miRNAs expressed in tumor compared to adjacent tissues, out of which hsa-miR-146a-5p was the most prominent overexpressed molecule in tumor cells. MiR-146a-5p regulates several important cellular pathways like Toll-like receptor, NF-κB, and ErB. Actually, miRNA-146a-5p targets almost 40 genes, one of them being the well-known *NRAS* gene (Aksenenko et al., 2019). Prognostic miRNA tissue signatures that match the recent ones were reported several years ago. Twelve-miRNA and 15-miRNA signatures were associated with stage III patients’ extended survival. Cross-validation between these two datasets revealed five miRNAs (miR-142-5p, miR-150-5p, miR-342-3p, miR-155-5p, and miR-146b-5p) that were highly and reproducibly associated with clinical patient prognosis (Jayawardana et al., 2016).

### Discriminating Melanoma From Other Skin Cancers Through miRNAs

Discriminating between benign and malign proliferation of melanocytes and discriminating between different skin cancers are any pathologist’s goals. As skin cancer has the highest frequency in humans, discriminating between non-melanoma skin cancers and melanoma is extremely important. A gene-specific intron retention signature was developed to differentiate melanoma from other skin cancers. Using RT-qPCR technology, total RNA was isolated from all types of skin cancer samples. Complex bioinformatics was performed, and the *c-MYC*, *SRPX2*, and *Sestrin-1* genes were demonstrated to undergo intron retention just in melanoma. miRNAs were generated for these genes, and thus this pattern could molecularly differentiate other skin cancers from melanoma (Giannopoulou et al., 2019).

Using RT-PCR, the miRNA expression profile was studied in melanocytic lesions, namely, benign nevi, cutaneous melanoma, and borderline melanocytic tumors. The authors show that, in melanomas, there are increased expressions for miR-21 and miR-155 compared to benign nevi. In borderline lesions, miR-21 and miR-155 were significantly overexpressed and associated with mitotic activity and thickness. miRNA expression can characterize atypical melanocytic proliferation (Grignol et al., 2011). One year later, using TaqMan^®^ RT-PCR assay, miR-21 was also studied by another group investigating dysplastic nevi and primary melanomas. MiR-21 was found increased in a continuous level from dysplastic nevi to melanomas and once more elevated in melanoma metastases. The expression level of miR-21 was found correlated with Breslow index, clinical stage, and shorter overall survival. Experimental *in vitro* antisense-mediated miR-21 inhibition reduced the tumor growth and induced apoptosis and therapy sensitivity through an increased Bax/Bcl-2 ratio (Jiang et al., 2012).

An earlier study comparing melanocytic nevi, melanoma tissue, and melanoma cell lines has shown that, in melanomas, miR-15b and miR-210 were significantly upregulated, while miR-34a was found significantly downregulated. In a clinical follow-up, only a high expression of miR-15b was associated with worse overall survival. It is one of the first studies that highlighted miRNA expression being different in malignant *versus* benign melanocytes (Satzger et al., 2010).

Using microarray analysis validated by qRT-PCR, improved molecular tissue markers were reported when three miRNAs (namely, miR-200c, miR-205, and miR-211) were found differentially expressed in primary compared to metastatic melanomas, these miRNAs acting like tumor suppressors (Xu et al., 2012). Using qRT-PCR for analyzing dysplastic nevi, in comparison to primary and metastatic melanomas, miR-106b expression was investigated. A high miR-106b expression was found correlated with Breslow index, tumor ulceration, clinical stage, and overall patient survival, pointing out that these miRNAs are independent prognostic factors for overall survival and patients’ risk stratification (Lin et al., 2015).

An outline of the main findings regarding miRNA expression in melanoma tissues and/or in the circulating form is presented in Table 2.

In cutaneous melanoma, besides the clear histopathological and clinical criteria stated by the American Joint Committee on Cancer (AJCC), such as tumor thickness, ulceration, mitoses, and lymph node and distant organ spread, new prognostic markers should be entered into this panel. Particular determinants like genetic mutation(s), particular epigenetic features, and the host’s immune response are important criteria for predicting patient outcomes (Neagu et al., 2013). Future validation studies for new epigenetic prognostic biomarkers should be expanded (Weiss et al., 2015).

### miRNA Involvement in Therapy Resistance in Cutaneous Melanoma

With the advent of targeted therapy in melanoma, namely, BRAF kinase inhibitors for *BRAF* mutant tumors, epigenetic studies emerged focusing on the processes that underlie therapy resistance. miRNomes and transcriptomes were studied in *in vitro* melanoma cellular models. The study revealed that particular miRNAs and genes are differently expressed in drug-resistant *versus* drug-sensitive cell lines (e.g. miR-92a-1-5p, miR-708-5p, and *DOK5* and *PCSK2* genes). In drug-resistant cell lines, it was shown that a low MITF/AXL ratio is regulated by miRNAs. Thus, the drug resistance process in melanoma therapy has particular sets of miRNAs that regulate particular genes that would lead to resistance (Kozar et al., 2017).

In combined therapies with BRAF and MEK inhibitors, therapy resistance occurs quite rapidly during therapy initiation. A low expression of miR-579-3p was found correlated with poor survival and with staging. Moreover, in melanoma cell lines that are resistant to BRAF/MEK inhibitors, this miRNA expression was reported as low. MiR-579-3p targets the 3′-UTR region of oncoproteins BRAF and E3 ubiquitin-protein ligase, MDM2. In tumor samples harvested before and after therapy resistance occurrence, miR-579-3p is strongly downregulated upon resistance installment (Fattore et al., 2016).

Recent reports have studied the involvement of miRNAs in the chemoresistance of melanoma cells, as it is known that this type of tumor lacks sensitivity to cytostatics. Using qRT-PCR and mouse xenograft assay, it was shown that miR-211 was found decreased. Bisulfite sequencing PCR technology indicated that DNA hypermethylation induced the downregulation of miR-211 in tumor tissues. Reversing the process, namely, the epigenetic modification that downregulates miR-211, chemosensitivity of melanoma cells can be achieved (Li et al., 2019). Resistance to MAK inhibitors was shown to be associated with another miRNA, namely, miR-214. Using RNA-seq analysis, it was demonstrated that melanoma cells frequently express β-catenin mRNA isoforms and lack a miR-214 target site. Therefore, using tandem miRNA and mRNA-seq analysis, new targets were identified for miR-214 action. The novel miR-214 targets were ankyrin repeat domain 6 (ANKRD6) and C-terminal-binding protein 1 (CTBP1), these being involved in the negative regulation of Wnt signaling. Mechanistically, the overexpression of miR-214/knockdown of ANKRD6 or CTBP1 increases the melanoma cell’s pro-tumorigenic characteristics, proliferation, and migration and decreases its sensitivity to MAPK inhibitors (Prabhakar et al., 2019).

#### miRNAs as Therapy Efficacy Markers

When following therapy efficacy in melanoma, in addition to the classical circulating markers as LDH, S100 calcium-binding protein B (S100B), melanoma inhibitory activity (MIA) (Neagu et al., 2009), and, lately, ctDNA (Seremet et al., 2019), circulating miRNAs gained interest. As circulating miRNAs have structural stability, they can become robust non-invasive biomarkers when several technicalities can be standardized, such as the sample type (plasma *versus* serum) and pre-analytical and analytical workflows (Mumford et al., 2018).

With the advent of clinical approval of immunotherapy in melanoma (Ancuceanu and Neagu, 2016), finding miRNA efficacy markers is an important goal. In melanoma patients, the accumulation of myeloid-derived suppressor cells with the phenotype CD14^+^HLA-DR^–^ (myeloid-derived suppressor cells, MDSCs) stands for the cells that can hinder immunotherapy efficacy. In a recent study, it was shown that a panel of miRNAs (e.g. miR-146a, miR-155, miR-125b, miR-100, let-7e, miR-125a, miR-146b, and miR-99b) is related to MDSCs and immune checkpoint inhibitor resistance. Actually, these miRNAs were found involved in the transformation of monocytes in MDSCs and in melanoma patients. These miRNAs were found increased in blood CD14^+^ cells, were identified circulating in plasma, and, in tumor tissues, were correlated with MDSC infiltrates. Various methods were implied by the group; thus, after RNA extraction from circulating monocytes, from extracellular vesicles, from melanoma cells, and from melanoma specimens using mirVana miRNA isolation kit, qPCR analysis was performed to evaluate the gene and miRNA expression levels.

Before immunotherapy installment (whether CTLA-4 or PD-1 blockade), the miRNA levels in patients’ plasma could indicate the future clinical efficacy of the immunotherapy. Thus, miRNAs that append to the circulating MDSCs can be validated further in blood markers for therapy efficacy (Huber et al., 2018).

Studies regarding responders *versus* non-responders to therapy in melanoma do not abound. Nevertheless, a study published in 2019 focusing on BRAF and MEK inhibitors has evaluated the miRNA profiling of melanoma patients at baseline and during resistance acquirement to therapy. The authors point out that restoring miR-126-3p expression in dabrafenib-resistant melanomas could increase the drug sensitivity of non-responders (Caporali et al., 2019).

It is highly probable that, in terms of finding new therapy efficacy markers and stratifying patients that would best benefit from a certain therapy, circulating miRNAs would gain future clinical importance.

### lncRNA – Regulators of miRNAs in Melanoma

Long ncRNAs (lncRNAs) have recently been recognized as important regulators of transcriptional, post-transcriptional, and translational processes. lncRNAs are non-coding RNAs (200 nt and 100 kb) with multiple functions as chromatin regulators, ribonucleoprotein scaffolds, regulators of transcription factors, and matrix for other post-transcriptional molecules such as miRNAs (Dinescu et al., 2019).

In melanoma, lncRNA ILF3-AS1 was found upregulated in both melanoma tumors and melanoma cell lines. The expression of this lncRNA was correlated with worse prognosis for melanoma patients. Experimental findings showed that ILF3-AS1 interacts with EZH2, promoting the link of EZH2 to the miR-200b/a/429 promoter, and, hence, represses miR-200b/a/429 expression. This negative correlation between ILF3-AS1 and miR-200b/a/429 was verified in melanoma tumor tissues. Functional tests have shown that, when lncRNA ILF3-AS1 is upregulated, several pro-tumorigenesis functions are increased – cell proliferation, migration, and invasion – processes that repress miR-200b/a/429. The authors point out that this tandem could become a new therapy focus in melanoma (Chen et al., 2017). Interestingly, another lncRNA was reported as having similar activity to ILF3-AS1. lncRNA-HEIH is also highly expressed in melanoma tissues, promotes tumor proliferation, migration, and invasion, and it binds to the miR-200b/a/429 promoter, repressing its transcription. Therefore, lncRNA-HEIH is another player in the miR-200b/a/429 expression involved in melanoma tumorigenesis and a prognostic epigenetic biomarker (Zhao et al., 2017).

A study published in 2019 showed an integrative analysis of a large group of epigenetic-related molecules, namely, lncRNA, miRNA, and mRNA, to design a competing endogenous RNA (ceRNA) network in melanoma. After complex analysis using data from The Cancer Genome Atlas (TCGA), the Gene Ontology (GO) database, and the Kyoto Encyclopedia of Genes and Genomes (KEGG), some epigenetic traits were found for metastatic melanoma. Overall survival was correlated with differentially expressed mRNAs, miRNAs (e.g. miR-29c, miR-100, miR-142-3p, miR-150, and miR-516a-2), and lncRNAs (e.g. AC068594.1, C7orf71, FAM41C, GPC5-AS1, MUC19, and LINC00402). It is remarked that the ceRNA network should be further developed to identify the best epigenetic pattern that can predict prognosis in melanoma patients (Wang et al., 2019).

As miRNAs regulate over 60% of human genes and since cutaneous melanoma has a high genetic heterogeneity, miRNA alterations, stable and detectable in tissue/body fluids, make them robust candidate biomarkers in melanoma (Varamo et al., 2017). Melanoma incidence is steadily rising in the last decades (Aftab et al., 2014); thus, melanoma molecular biology features should be thoroughly studied. miRNAs are probably key players in melanogenesis as inducers and blockers. Already, miRNA expression profiling with a focus on some sets was identified as linked to tumor proliferation, migration, and invasion, but is also correlated to apoptosis induction and generation of an anti-tumoral immune response (Ross et al., 2018).

## miRNAs in non-Melanoma Skin Cancers

### Cutaneous Squamous Cell Carcinoma

In non-melanoma skin cancers, the majority of published work regarding miRNA involvement in the diagnosis/prognosis focuses on cutaneous squamous cell carcinoma (CSCC). CSCC has a rising tendency and is the second most frequent cancer in humans. In mouse cancer cell lines, miRNAs were identified, and in these studies, the same miRNA panel was searched for in human tumors. MiR-205 and miR-203 were selected and matched the CSCC clinical prognosis. Thus, miR-205 was associated with desmoplasia, perineural invasion, and infiltrative pattern, all these features being known to be associated with poor prognosis. MiR-205 was associated clinically with local recurrence and poor prognosis. In contrast, miR-203 was expressed in tumors that displayed tissue characteristics associated with favorable prognosis. Therefore, the authors point out that, in CSCC, miR-205 and miR-203 expressions are mutually exclusive, pinpointing that these molecules can have prognostic potential (Canueto et al., 2017). Another miRNA recently found overexpressed in CSCC samples is miR-221. Functional tests have shown that *PTEN* is the direct target gene for miR-221. Thus, in CSCC, miR-221 has an oncogenic function by interacting with *PTEN*. Moreover, future anti-miR-221 can be developed for CSCC diagnosis and treatment (Gong et al., 2019).

An earlier study focused on miR-20a as a prognostic biomarker in CSCC. Using qRT-PCR applied to over 150 CSCC tissues and compared to healthy adjacent normal tissues, it was shown that miR-20a expression was lower in CSCC tissues compared to normal tissues, and this expression was correlated with the TNM stage. Moreover, these findings showed that patients had a significantly worse overall survival when miR-20a expression was found upregulated in comparison to patients with high tissue expression. This tissue molecule can become an independent prognostic biomarker of CSCC and, in the future, can even enter the treatment armamentarium (Zhang et al., 2015).

An extensive review published in 2019 has shown that, in CSCC, there is an array of miRNAs with oncogenic functions while other miRNAs are tumor suppressors. Therefore, families of tumor inductors that were reported in CSCCs comprise a large set of miRNAs (e.g. miR-21, miR-205 miR-365, miR-31, miR-186, miR-142, and miR-135b) that act generally on *PTEN*, *PDCD4*, *GRHL3 HOXA9*, and *RhoBTB*, these genes being involved in seminal pro-tumorigenic processes like tumor growth, invasion, migration, maintenance of stem cell properties, and hindrance of apoptosis. From the tumor suppressor family of miRNAs (e.g. miR-34a, miR-125b, miR-181a, miR-148a, miR-20a, miRNA-203, miR-204, miR-199a, miR-124, and miR-214), there are members that regulate genes like HMGB1, SIRT6, MMPs, MAP kinases, KRAS, LIMK1, c-MYC, SHP2, CD44, BCAM, FZD6, DDR1 and ERKs. Their action is to regulate processes like cell cycle, epithelial–mesenchymal transition, and stemness while promoting cellular apoptosis and senescence (Garcia-Sancha et al., 2019).

### Cutaneous Lymphoma

Cutaneous lymphoma, a non-Hodgkin’s lymphoma subtype, is triggered by abnormal B or T cells. Cutaneous T cell lymphoma (CTCL) and cutaneous B cell lymphoma (CBCL) induce alterations in the skin and several other sites (e.g. lymph nodes, peripheral blood, and internal organs). CTCL is the most common type, covering up to 80% of all cases of cutaneous lymphomas (Willemze et al., 2005). Recently, CTCL was entered into miRNA guided therapy because CTCL progression is characterized by aberrant miRNAs (Kohnken and Mishra, 2019). Mycosis fungoides (MF), the most common form of CTCL, has around 30% of patients diagnosed with an aggressive form (Willemze et al., 2005).

DICER, the ribonuclease III-like enzyme that processes miRNA, was investigated in various CTCL forms. Out of all the types, DICER was proven by immunohistochemical analysis to be a negative predictive factor in MF patients with no correlation with gender, histological subtype, primary localization, age, and recurrence. The note that the authors pointed out in this study published in 2011 was that “miRNA(s) might be of clinical relevance in CTCL” (Valencak et al., 2011). Just 1 year after this publication, another group showed in MF biopsies harvested from early and advanced stages that the assertion regarding miRNAs is correct. The group of Maj has identified, after isolation, reverse transcriptase reactions, and cDNA amplification, a panel of miRNAs: miR-15a, miR-16, miR-155, let-7a, let-7d, and let-7f. MF was characterized by a miR-155 overexpression, and metastatic MF was found with lower concentrations of let-7a, let-7d, and let-7f. Moreover, the level of let-7a expression proved to be an independent prognostic indicator (Maj et al., 2012).

In 2019, evaluating miRNA machinery genes (e.g. Dicer and Drosha) in MF patients, interesting new data were obtained. Low Drosha expression seems to be an independent predictor biomarker for advanced stages. Moreover, this expression was associated with lymphoma-specific death, the authors pointing out the tumor suppressor gene function of Drosha (Gambichler et al., 2019).

In 2018, utilizing a RT-qPCR platform, the miRNA expressions in tumor samples harvested from over 150 patients with early-stage MF were analyzed. A three-miRNA classifier was developed comprising miR-106b-5p, miR-148a-3p, and miR-338-3p, which correlated with clinical progression. Furthermore, this panel could stratify patients into high- and low-risk groups. This miRNA-based classifier has a good prognostic value and can further be used to orient patients’ therapy (Lindahl et al., 2018).

**FIGURE 2:**
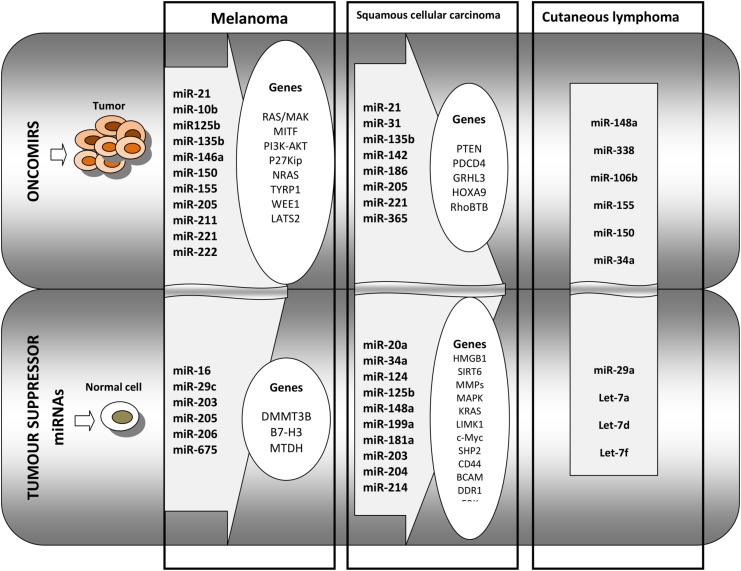
Overview of the main miRNAs and the regulated genes that are identified in melanoma, in squamous cell carcinoma, and in cutaneous lymphoma. The **first panel** of oncomirs represents the miRNAs that regulate pro-tumoral genes in melanoma, squamous cell carcinoma, and in cutaneous lymphoma. The **second panel** represents tumor suppressor miRNAs regulating genes that are involved in anti-tumoral processes in squamous cell carcinoma and in cutaneous lymphoma.

Cutaneous B cell lymphoma, although rarer, has a high relapse rate, up to 40%; thus, identification of patient groups most likely to relapse is of utmost importance. Using RT-PCR, 11 miRNAs were identified as being associated with the differentiation stage of B cells. From this set, miR-150 was found overexpressed in CBCL-type primary cutaneous marginal zone B cell lymphomas compared to the other CBCL type, centrofollicular lymphoma. Low levels of miR-155 and miR-150 were found associated with shorter progression-free survival in primary cutaneous marginal zone B cell lymphomas type. Thus, miRNA tumor analysis can aid in the diagnosis and prognosis of CBCL (Monsalvez et al., 2013). An overview of miRNAs functioning as tumor activating or tumor suppressors in melanoma, squamous cell carcinoma and cutaneous lymphoma is presented in Figure 2.

### Merkel Cell Carcinoma

Merkel cell carcinoma (MCC) is a rare tumor that develops in the skin. Development in elderly Caucasian males is frequent, in mostly immunocompromised individuals, where it has an aggressive evolution with >30% of deaths. Merkel cells that reside in the basal epithelial layer are subjected to malignant transformation, probably induced by Merkel cell polyomavirus infection. Discovered in 2008, Merkel cell polyomavirus was shown to be expressed in over 90% of MCC (Feng et al., 2008). MCC therapy comprises excision, chemo-, and radiotherapy, and, more recently, some Merkel cell polyomavirus vaccination (Zeng et al., 2012). However, local recurrence and early metastasis are registered in spite of the installed therapies. Novel therapies using antisense oligonucleotides or miRNAs that can regulate Atonal homolog 1 (*ATOH1*) expression, a tumor suppressor gene, could bring new clinical approaches in this disease (Krejci et al., 2010). Indeed, in 2019, new data emerged; thus, *ATOH1* was proven to bind and further activate miR-375, the highest abundant miRNA in MCC. In experimental models, it was shown that *ATOH1* knockdown in cell lines drastically reduced miR-375 expression. *ATOH1* overexpression induced a more metastasizing pattern in cell lines and an increased miR-375 expression. Testing Merkel cell polyomavirus infection in this model, it was demonstrated that the infection induces carcinogenesis *via* the induction of *ATOH1*. This approach can be developed further in a therapy target for MCC (Fan et al., 2019).

## Conclusion

Skin cancers, with their steadily increasing incidence, and with melanoma being one of the deadliest forms of cancer, need constant attention regarding the discovery of molecular mechanisms that govern tumorigenesis, metastasis, therapy resistance, and, last but not least, the development of new therapeutic strategies.

In the molecular events that characterize these complex processes, miRNAs, small non-coding RNAs that control gene expression at the post-transcriptional level, are recently gaining increased importance. Moreover, miRNA expression can constitute an important diagnosis/prognosis and therapy monitoring markers. As miRNAs have high stability and can be identified in circulation as well, their expression profiles in body fluids can reflect the progression status, and therefore their quantification can be developed in biomarkers with prognostic value. Using circulating miRNAs should overcome some issues regarding sensitivity and specificity, and probably in the future, additional epigenomic platforms could overcome these bottlenecks.

However, the involvement of miRNAs in non-melanoma skin cancer is less characterized concerning tumor initiation, metastasis, and progression stages; hence, a miRNA signature should also be further developed in diagnostic and prognostic biomarkers.

As miRNAs have gained a role in the substantial armamentarium of biomolecules regarded as disease markers, the need for implementing a reliable technology for assessing miRNAs, especially in clinic workflow, is mandatory. Current technologies comprise qPCR or microarray platforms, but these are still challenging due to high costs and the personnel expertise required. New hopes come from classic concepts transposed into a new perspective technology, such as miREIA which has the potential to be clinic-friendly besides its high sensitivity and specificity of detection.

In the epitranscriptomic domain, the research done recently has shown that miRNAs can have dual functions: pro- or anti-tumoral activity; therefore, their thorough evaluation is more so important for establishing accurate diagnostic and/or prognostic molecular markers in skin cancers.

## Author Contributions

MN conceived and designed the manuscript and wrote the sections “Main Technologies That Are Used for miRNAs Identification From Biological Samples” and “miRNAs Detected in Cutaneous Melanoma” of the manuscript. SC collected the data and wrote the abstract and the section “Introduction” of the manuscript. CC conceived the manuscript and was in charge of the overall direction and planning, and wrote the section “miRNAs Detected in Cutaneous Melanoma” of the manuscript. SZ wrote the sections “miRNAs in Non-melanoma Skin Cancer” and “Conclusion” of the manuscript. MN and SC organized the sections of the manuscript and revised the final form of the manuscript.

## Conflict of Interest

The authors declare that the research was conducted in the absence of any commercial or financial relationships that could be construed as a potential conflict of interest.
